# Metastatic Leptomeningeal Carcinomatosis From Primary Lobular Breast Cancer Found in a Medical School Cadaveric Dissection

**DOI:** 10.7759/cureus.44533

**Published:** 2023-09-01

**Authors:** Mary C Mitchell, James Pollock, Mary B Downs, David Stephen

**Affiliations:** 1 Medical School, Edward Via College of Osteopathic Medicine, Auburn, USA; 2 Anatomy, Edward Via College of Osteopathic Medicine, Auburn, USA; 3 Pathology, Edward Via College of Osteopathic Medicine, Auburn, USA

**Keywords:** cadaveric dissection, e-cadherin expression, lobular breast cancer, breast cancer pathology, leptomeningeal carcinomatosis (lc)

## Abstract

Leptomeningeal carcinomatosis (LC) is an uncommon sequelae of metastatic cancer affecting the pia and arachnoid mater. It has been postulated that recent improvements in cancer patient survival time have increased the frequency of LC and other rare metastatic conditions that patients previously would not have lived long enough to experience. LC carries a universally poor prognosis with a mean survival of between two to four months if treated; however, the recent increase in incidence has allowed for further research into the condition and potential treatments. Options for administering chemotherapy have been limited in the past, but recent developments in surgical chemotherapeutic ports have allowed for intrathecal delivery of drugs like methotrexate without systemic exposure. In fact, innovative delivery systems undergoing clinical trials can deliver these drugs in a metronomic fashion to limit the leukoencephalopathy complications of methotrexate. Primary breast cancer is the most common source of metastatic leptomeningeal lesions, and such a lesion was observed by the authors in the cadaver of a 70-year-old Caucasian female with unspecified breast cancer in a medical school anatomic laboratory. The cause of death was listed as “complication of malignant neoplasm of unspecified site of unspecified female breast.” Through this case report, we seek to develop our understanding of this rare metastatic phenomenon and highlight the importance of student cadaveric dissection.

## Introduction

Leptomeningeal carcinomatosis (LC) is a secondary cancer that occurs through either hematogenous spread or direct extension from a primary solid tumor, most often breast cancer. This cancer affects the pia mater, arachnoid mater, and subarachnoid space [1]. LC can also occur via direct extension from contact with brain metastases [2]. Lymphoma, breast carcinoma, especially its lobular variant, and melanoma are the three primary cancer types that lead to LC. While breast cancer is the greatest solid tumor at risk of LC [3], it is a rare presentation with an incidence of 5% [1]. Within this group, breast cancer patients with brain metastasis are at an increased risk of LC compared to breast cancer patients without brain metastases [1]. The primary breast cancers that pose the greatest risk for LC are the lobular histological subtype [2] and the triple-negative molecular subtype [1]. While lobular breast cancer is significantly less common than ductal, constituting less than 15% of all epithelial breast cancers, the loss of the epithelial-cadherin (E-cadherin) complex inherent to the lobular subtype seems to play a role in its preference for leptomeningeal metastasis [1,4]. A similar pathology of E-cadherin has been observed within specimens of gastric signet ring cell carcinoma, which also demonstrates a metastatic predilection for the leptomeninges [5,6]. A new onset of headache is the most common symptom that prompts providers to suspect LC metastases. However, patients can also present with other central nervous system (CNS) manifestations related to the location of dissemination, including cranial nerve palsies, visual field defects, numbness or tingling of the extremities, and cauda equina syndrome [1]. The diagnosis of LC metastasis is made through the demonstration of tumor cells on cerebrospinal fluid (CSF) examination, such as aspiration and cytology. T1-weighted magnetic resonance imaging (MRI) can also be used to visualize LC metastasis. This will show enhancement of the sulci, cranial nerve roots, or distinct nodules. However, lumbar puncture with CSF analysis conducted three times is preferred, as it is found to be 90% sensitive [1]. Treatment is personalized for each patient and depends on the patient’s functional status and extent of metastases. Intrathecal chemotherapy, systemic chemotherapy, and radiation may be used. Intrathecal (CSF) chemotherapy and systemic chemotherapy are used to prolong patients’ lives, but they are not typically performed with curative intent [7]. Conversely, radiotherapy is used to reduce neurological symptoms and allow for better CSF flow in patients with CSF flow obstruction or other neurological symptoms [1]. However, in some cases, due to the aggressive course and poor prognosis, some clinicians transition to palliative care with the diagnosis of LC. The one-year survival rate of LC from primary breast cancer is 16%, with the time of diagnosis of LC to death averaging 18 weeks [8].

## Case presentation

A leptomeningeal lesion was discovered while conducting a prosection on a cadaver of a 70-year-old Caucasian female with breast cancer of an unspecified histologic subtype. The donor program listed her cause of death as a “complication of malignant neoplasm of unspecified site of unspecified female breast.”

**Figure 1:**
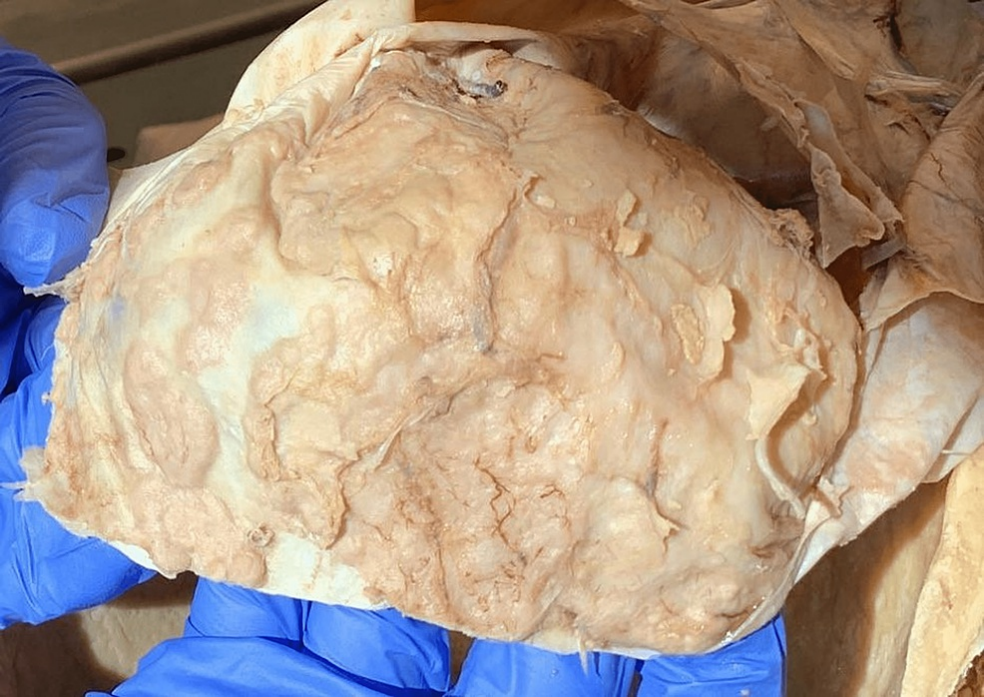
Leptomeningeal metastasis

**Figure 2:**
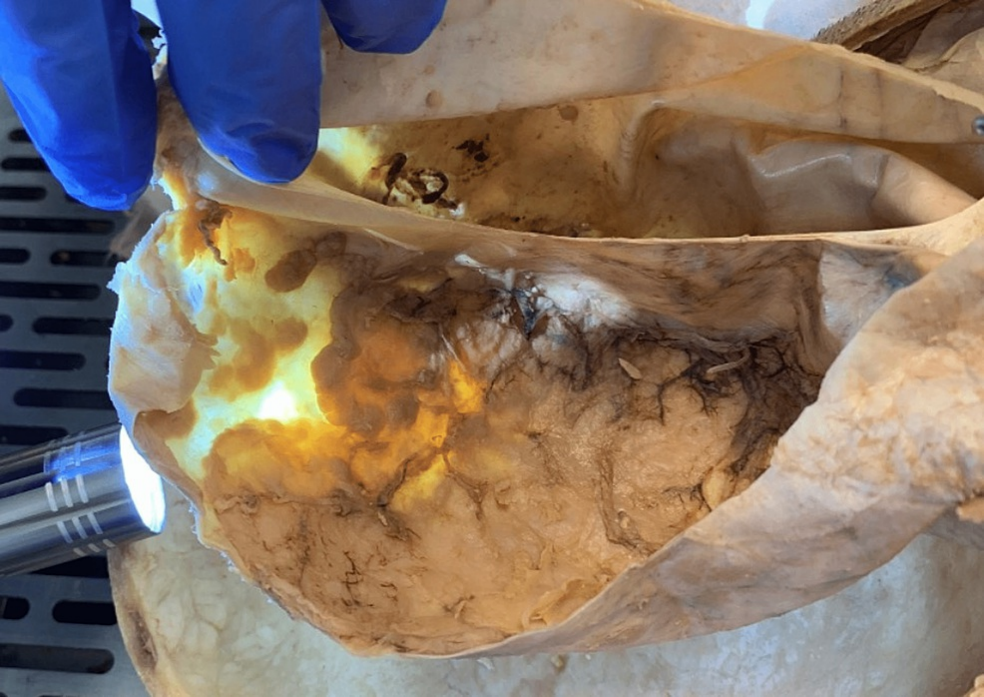
Transillumination of leptomeningeal metastasis

While attempting to isolate the brain, removal of the calvaria and reflection of the dura mater was considerably difficult as these individual components appeared adherent to the underlying structures. Once the bony components were removed, an incision was made into the superior dome of the dura mater, and the meninges reflected. Upon reflection, a series of geographical lesions were noted on the deep surface of the meninges overlying both hemispheres of the frontal lobe (Figures 1-2). It could not be determined from gross examination if the lesion involved the dura mater. The lateral ventricles of the brain appeared to be of normal size upon coronal sectioning, suggesting that there were no hydrocephalic complications of the lesion.

It was striking to find what appeared to be a metastatic breast cancer lesion involving the leptomeninges and not the brain parenchyma. Samples of the lesion were obtained for hematoxylin and eosin (H&E) stain and submitted for histological examination, confirming the preexisting diagnosis of metastatic carcinoma. We expected to confirm this secondary metastasis was related to carcinoma of the breast through the demonstration of ductal or lobular histologic features. Ductal carcinoma grows in tubular structures, while lobular carcinoma is defined by lines of atypical cells [9]. Ductal carcinoma is a more common subtype than lobular carcinoma; however, lobular carcinoma has been shown to metastasize to the leptomeninges more commonly than ductal carcinoma [1,8].

**Figure 3:**
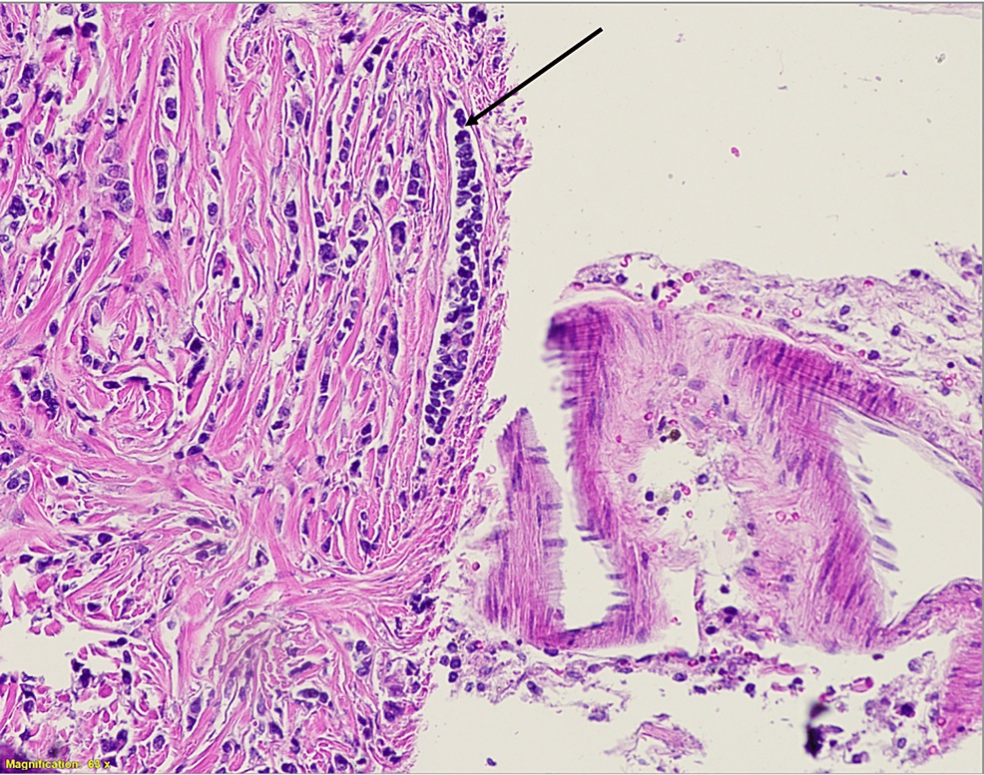
Leptomeningeal metastasis H&E stain at 63X magnification (view 1)

**Figure 4:**
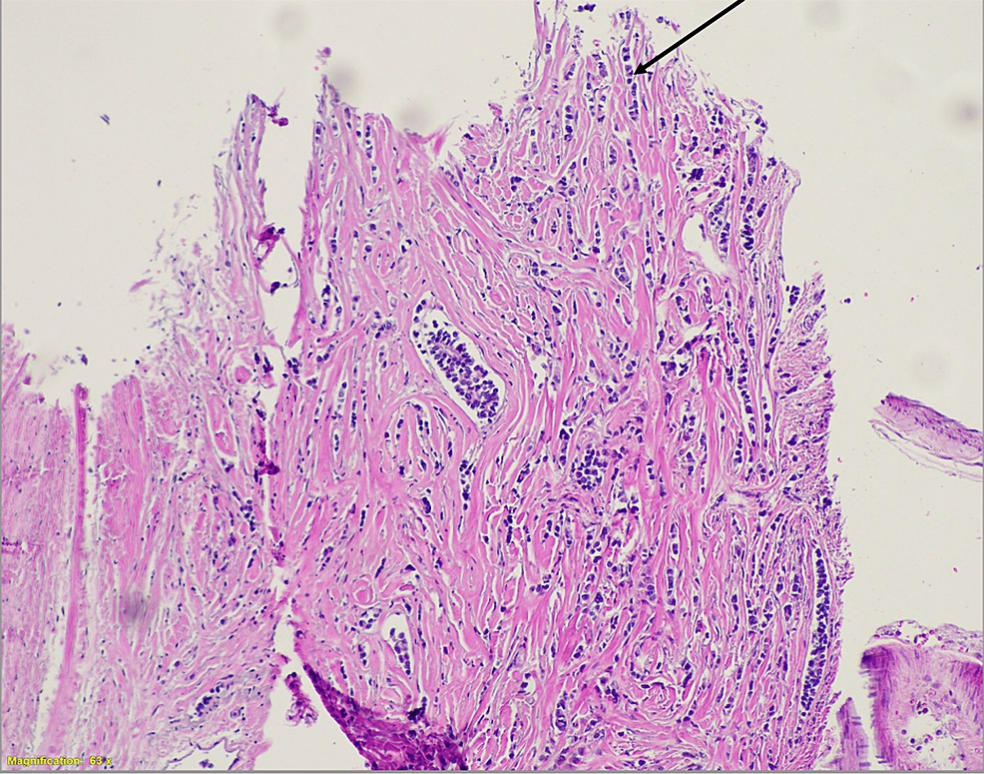
Leptomeningeal metastasis H&E stain at 63X magnification (view 2)

Upon microscopic examination of the H&E stains of the metastases, we visualized numerous rapidly dividing basophilic cells infiltrating the arachnoid mater. These cells were largely arranged in a linear fashion (Figures 3-4). This pattern was consistent with lobular carcinoma, confirming the leptomeningeal metastasis was from the breast and was indeed the lobular subtype based on the prior established pathological diagnosis of invasive lobular carcinoma.

## Discussion

Leptomeningeal metastasis from primary breast cancer is commonly associated with the lobular histological tumor subtype. While primary lobular breast cancer is less common than primary ductal breast cancer, it is more commonly associated with meningeal metastasis due to its loss of the E-cadherin [1]. The E-cadherin regulatory protein is a transmembrane glycoprotein that allows for cell-cell adhesion, creating tissue continuity [10]. When E-cadherin is absent, cell-cell adhesion becomes compromised. This creates a path for cellular dissemination from the primary tumor [1]. Therefore, lobular breast cancer can be differentiated from ductal carcinoma on H&E staining by atypical cells arranged in a linear pattern [9], as opposed to ductal carcinoma, which appears in well-defined tubular structures. The loss of cell-cell adhesion dictates the behavior of this cancer. As lobular carcinoma shows a different pattern of metastatic spread than its ductal counterpart, it is more commonly associated with bilateral breast involvement and leptomeningeal metastasis [1]. Clinically, breast cancer histology is important to determine surgical treatment, as recommendations differ with each subtype. This evaluation can be done through breast biopsy when determining receptor status and risk assessment through molecular testing or immunohistochemical markers. Ductal carcinoma patients are more likely to be responsive to neoadjuvant chemotherapy and breast-conserving surgery, depending on tumor node metastasis (TNM) staging [11]. However, patients with lobular breast cancer are more likely to undergo mastectomy as lobular breast cancer is less responsive to neoadjuvant chemotherapy and more prone to positive surgical resection margins with breast-conserving surgery [11]. 

Pathologic staging is an important element that guides a patient’s treatment course and provides the best estimates of their disease prognosis. The patient, in this case, was described in the context of cadaveric dissection; however, there is still educational merit in considering the stage of their lobular breast cancer and what treatment would be recommended had the patient been able to receive appropriate therapy. The American Cancer Society has developed a TNM staging system for breast cancer that can be applied for such approximations, with the system itself last being revised in 2021 [12]. Because the patient in this case had already undergone unilateral radical mastectomy, it is unlikely that the primary breast tumor could be accessed. Therefore, the patient’s disease is designated as TX. Likewise, the patient’s prior mastectomy makes accessing nearby lymph nodes impossible, so her disease is identified as NX. The presence of leptomeningeal metastases, however, indicates a pathological staging of M1 and a clinical stage of IV. With advanced disease and no expectation for a cure, chemotherapy and hormone therapy are considered the primary treatment modalities, pending hormone sensitivities, with surgery and radiation therapy serving as options for symptomatic management [13].

More specifically, it has been previously discovered that the most effective chemotherapy regimen for leptomeningeal disease consists of high-dose methotrexate with a rescue dose of folic acid to minimize the adverse effects of folate deficiency, such as neutropenia and leukoencephalopathy [14]. For patients receiving long-term chemotherapy, the use of the Ommaya reservoir system and other port devices has made the delivery of chemotherapeutic drugs to the interventricular system more effective than systemic methods [3]. The use of intrathecal chemotherapeutic devices, however, may be complicated by CSF leakage and a significant increase in intracranial pressure. The incidence of these complications may be reduced with a metronomic biofeedback pump that monitors both drug concentration and intraventricular pressure while delivering chemotherapy. The active monitoring of intrathecal methotrexate concentration could also limit the previously discussed complications of methotrexate toxicity. One such pump is currently undergoing clinical trials [2,15]. Though an uncommon culprit for leptomeningeal metastases, HER2+ carcinoma has shown a clinical response to trastuzumab and capecitabine combination therapy by way of improved neurological symptoms in LC patients. Trastuzumab binds HER2 and limits proliferation in dependent cancers, while capecitabine is a prodrug of fluorouracil that inhibits DNA synthesis. Trastuzumab is also thought to facilitate antibody-mediated cellular toxicity, leading to the death of malignant cells expressing HER2 [16]. These patients appeared to respond to systemic chemotherapy because their extensive leptomeningeal metastases disrupted the blood-brain barrier [17].

This rare presentation certainly explains the donor’s cause of death and serves as an example of the value of student cadaver labs. During an era of medical education that, at least partially, has shifted toward online modules and virtual experiences, it is important to consider the incorporation of rare diseases or presentations within a program’s coding. Indeed, the sort of pathological variety exemplified by this case demonstrates the level of diversity and novelty exhibited by an in-person cadaveric study that is simply difficult to account for amid virtual lab experiences that are still in their growth and development stages.

## Conclusions

LC, while rare in patients with primary breast cancer, is a late-stage disease with a poor prognosis and merits appropriate education for medical professionals to optimize what few treatment options there may be for these patients. The opportunity to uncover this presentation of lobular breast cancer in a medical school cadaveric lab offers a unique, yet valuable, insight into this disease state and should be viewed as both a vote of confidence on the value of early exposure to high-fatality diseases and in-person cadaveric lab experiences. This case highlights the importance of in-person cadaveric dissections in medical education as they often reveal pathological findings both related and unrelated to the cause of death, which are omitted from virtual lab experiences.
